# *Heyndrickxia coagulans* strain SANK70258 suppresses symptoms of upper respiratory tract infection via immune modulation: a randomized, double-blind, placebo-controlled, parallel-group, comparative study

**DOI:** 10.3389/fimmu.2024.1389920

**Published:** 2024-06-17

**Authors:** Masanori Aida, Naoyuki Togawa, Kazuyuki Mizuyama, Yoshinori Aoki, Shouhei Suehiro, Akiho Sakamoto, Noriyoshi Uchida, Ryouichi Yamada

**Affiliations:** ^1^ Science & Innovation Center, Mitsubishi Chemical Corporation, Yokohama-shi, Kanagawa, Japan; ^2^ Department of Internal Medicine, Doujin Memorial Foundation Meiwa Hospital, Tokyo, Japan; ^3^ Food & Healthcare Deptartment, Mitsubishi Chemical Corporation, Tokyo, Japan

**Keywords:** Heyndrickxia coagulans strain SANK70258, Weizmannia, Bacillus, plasmacytoid dendritic cells, IFNα, butyric acid, anti-inflammatory, upper respiratory tract infection

## Abstract

Probiotic consumption strongly influences local intestinal immunity and systemic immune status. *Heyndrickxia coagulans* strain SANK70258 (HC) is a spore-forming lactic acid bacterium that has immunostimulatory properties on peripheral tissues. However, few reports have examined the detailed effectiveness of HC on human immune function and its mechanism of action. Therefore, we conducted a randomized, double-blind, placebo-controlled, parallel-group study to comprehensively evaluate the effects of HC on immunostimulatory capacity, upper respiratory tract infection (URTI) symptoms, and changes in intestinal organic-acid composition. Results of a questionnaire survey of URTI symptoms showed that runny nose, nasal congestion, sneezing, and sore throat scores as well as the cumulative number of days of these symptoms were significantly lower in the HC group than in the placebo group during the study period. Furthermore, the salivary secretory immunoglobulin A (sIgA) concentration was significantly higher, and the natural killer (NK) cell activity tended to be higher in the HC group than in the placebo group. In addition, we performed an exposure culture assay of inactivated influenza virus on peripheral blood mononuclear cells (PBMCs) isolated from the blood of participants in the HC and placebo groups. Gene-expression analysis in PBMCs after culture completion showed that IFNα and TLR7 expression levels were significantly higher in the HC group than in the placebo group. In addition, the expression levels of CD304 tended to be higher in the HC group than in the placebo group. On the other hand, the HC group showed a significantly higher increase in the intestinal butyrate concentration than the placebo group. HC intake also significantly suppressed levels of IL-6 and TNFα produced by PBMCs after exposure to inactivated influenza virus. Collectively, these results suggest that HC activated plasmacytoid dendritic cells expressing TLR7 and CD304 and strongly induced IFNα production, subsequently activating NK cells and increasing sIgA levels, and induced anti-inflammatory effects via increased intestinal butyrate levels. These changes may contribute to the acquisition of host resistance to viral infection and URTI prevention.

## 1 Introduction

The intestinal tract of a healthy individual contains more than 100 trillion intestinal bacteria of 1000 species (1). The balance of this diverse intestinal microbiota influences the health of the host, and probiotics have long been used as a means to achieve this balance (1, 2). Probiotics are defined as live microorganisms that provide health benefits to the host when ingested in appropriate amounts, and some bacterial strains have immunostimulant effects through the produced metabolites and bacterial components and the modification of the intestinal microbiota composition (3–5). The immunomodulatory and antiviral effects of probiotics have attracted increasing attention in recent years owing to the global threat of pandemic viruses and the emergence of drug-resistant bacteria from antibiotic overuse (6, 7). Probiotics have been reported to improve the symptoms of upper respiratory tract infection (URTI) through the activation of natural killer (NK) cells and increased secretion of immunoglobulin A (IgA) (8–11). The activation of plasmacytoid dendritic cells (pDCs) and the associated enhancement of IFNα production has long been a target for NK cell activation and enhanced IgA production. Exposure of inactivated influenza virus to peripheral blood mononuclear cells (PBMCs) from humans who ingested with *Lactococcus lactis* ssp. *lactis* JCM5805 has been reported to strongly enhance the expression of genes related to IFNα in PBMCs (12). This study also reported the possibility of reducing URTI and influenza symptoms via pDC activation. *Gluconacetobacter hansenii* GK-1, an acetic acid-producing bacterium, is reported to contribute to pDC activation and induce an increase in secretory IgA (sIgA) production and NK cell activity (13). Therefore, an antiviral response via activation of pDCs by lactic acid-producing or acetic acid-producing bacteria has been suggested; however, these reports were verified using dead bacteria, and reports using probiotics are lacking.

*Heyndrickxia* (syn. *Weizmannia*, *Bacillus*) *coagulans* SANK70258 strain (HC) is a lactic acid-producing bacteria that was isolated by Nakayama et al. in 1949 (14). HC is a spore-forming probiotic, with effects such as improving bowel movements (14). Several clinical trials of HC have been conducted, with one study reporting that the administration of a symbiotic combining this strain and galactooligosaccharides reduced fecal p-cresol content and improved skin roughness and another reporting that HC supplementation increased the number of *bifidobacteria* in the intestine, decreased intestinal pH, and increased the frequency of bowel movements, demonstrating the various efficacy and safety effects of HC in humans (15, 16). Furthermore, reports related to the immunostimulatory properties of HC exist, including a report that HC administration improves seasonal allergic rhinitis and a report that the production of IL-10, an anti-inflammatory cytokine, is enhanced by the ingestion of a multibiotic containing HC (17, 18). *In vivo* and *in vitro* evaluations have shown that HC can strongly induce IgA production and NK cell activation locally in the intestinal tract and peripheral tissues (19). A study of a different strain has shown that ingestion of *H. coagulans* increases NK cell activity (20). In addition, the enhancement of intestinal immune function by HC via modification of the intestinal microbiota is reported to contribute to the acquisition of resistance to infection by pathogenic protozoa in livestock and ameliorate weight loss caused by infection (21, 22). Several reports have suggested that HC may have immunostimulatory properties; however, few reports have examined the detailed effectiveness of HC on human immune function and its mechanism of action.

Therefore, we conducted a randomized, double-blind, placebo-controlled, parallel-group study to comprehensively evaluate the effects of HC on immunostimulatory capacity, URTI symptoms, various clinical markers, and changes in intestinal organic acid composition.

## 2 Materials and methods

### 2.1 Tests food and groups

A capsule containing 67 mg of *H. coagulans* SANK70258, equivalent to 1 billion colony-forming units of lactic acid bacteria, was used as the test food. A capsule with the same appearance as the test capsule but with no active ingredients was used as the placebo capsule (**Supplementary Table 1**). Two experimental groups were established: one group received the test capsule with HC and the other group received the test capsule without HC (Placebo).

### 2.2 Participants

This study was conducted in accordance with the tenets of the Declaration of Helsinki, was approved by the Research Ethics Committee of the Medical Station Clinic (approval date: December 24, 2020; approval number: 20000022), and was registered and published in the UMIN clinical trial registration system (clinical trial registration number: UMIN000042937). The study participants received a full explanation of the study contents from the investigator and provided voluntary written consent to participate.

Participants who met the following inclusion criteria were included: (1) healthy working men and women aged between 20–65 years; (2) with a preference for those with low salivary sIgA at baseline; (3) those who, in a self-reported survey, were more likely to have had a cold or flu in the past two years.; (4) those who have had a URTI in the last two winters; and (5) those who have been fully informed of the purpose and details of the study, have the ability to give consent, and have voluntarily agreed to participate in the study based on a thorough understanding of its purpose and details.

In addition, the following exclusion criteria were established: (1) those who have, are receiving treatment for, or have a history of diabetes, renal, hepatic, cardiac, or other serious diseases, thyroid disease, adrenal disease, or metabolic diseases; (2) those who have chronic diseases and regularly use medications; (3) those who cannot stop taking foods containing lactic acid bacteria, *bifidobacteria*, oligosaccharides, or viable organisms during the study period; (4) those who are regularly taking supplements or health foods that may affect immunity; (5) those who are undergoing treatment that may affect the test results; (6) those who cannot abstain from alcohol for two days prior to each test date; (7) those who may develop allergies related to the study; (8) those who have a history of digestive disease or surgery that may affect digestion and absorption; (9) those who have a history of abnormal laboratory values or cardiopulmonary function that may affect participation in this study; (10) those who have current or a history of drug or alcohol dependence; (11) those who are prone to diarrhea when consuming dairy products; (12) those who work day and night shifts or engage in physical labor such as heavy lifting; (13) those who have a history of heart disease that may affect participation in this study; (14) those who have undergone or will undergo dental or oral treatment from one month prior to the pre-test or during the study period; (15) those with dental or oral abnormalities that cause bleeding; (16) those with physical measurements, physical examination values, and clinical examination values that are outliers of the reference range before the start of the study; (17) those who are enrolled in other clinical trials at the start of this study and those who intend to enroll during the study period; (18) those who intend to lactate or become pregnant during the study period; (19) those who are deemed unsuitable as participants based on their responses to the lifestyle questionnaire; and (20) those who are deemed unsuitable as participants by the treating physician.

The study food management was conducted as follows: (1) research institutions supplied research food to contract research organizations, and contract research organizations supplied research food to medical facility support organizations; (2) the medical facility support organization sent the required amount of research food to the participants; and (3) any excess research food after research completion was collected by the medical facility support organization and returned to the contract research organization, who subsequently returned it to the research organization.

The following restrictions on the participants were applied: (1) participants were instructed to not change their lifestyle habits, including eating, drinking, exercising, and sleeping, during the study period from those before the study as much as possible; (2) participants were instructed not to engage in excessive exercise, moderation, or overeating during the study period that deviated significantly from their daily range; (3) participants were instructed not to start a new exercise routine or change their previous exercise habits during the study period; (4) participants were instructed not to receive any vaccinations, such as the flu vaccine, during the study period; (5) participants who used any medication were instructed to record the name of the product and the amount used in a daily logbook; (6) participants were instructed to consume the prescribed amount of research food each day and record the time of consumption in a daily logbook; (7) participants were asked to keep a daily diary for the specified period; (8) participants were instructed to go to bed by 12:00 p.m. on the day before the examination to obtain adequate sleep; and (9) participants were asked to brush their teeth without toothpaste or mouthwash on the day of the examination at least 1 h before the examination.

Participants were prohibited from the following: (1) the use of dietary supplements and other health foods was prohibited during the study period; (2) participants were fasted from the time they woke up until the end of the examination on the day of the study (drinking water was allowed), and they had to confirm that they had fasted for at least 8 h from the night before the study; (3) the use of medication was prohibited on the day before the examination visit; (4) participants were forbidden from drinking alcohol for two days before the examination visit; (5) participants were prohibited from strenuous exercise on the day before the examination until the end of the examination on the day of the examination; and (6) smoking was prohibited from the day of the examination until the completion of the examination.

The sample size was determined based on the number of cases reported in a human study that investigated the effects of HC on immunostimulatory effects in the eye (17). The required sample size was > 21 participants when α was 0.05 and power (1-β) was 80%. G*Power ver3.1.9.2 (Heinrich-Heine-Universitat, Düsseldorf, Germany) was used to calculate the sample size. In this study, the sample size for efficacy analysis was 79 (39 and 40 participants in the HC and placebo groups, respectively).

### 2.3 Study schedule

This study was a randomized, placebo-controlled, double-blind, parallel-group comparison study conducted from December 2020 to July 2021. Participants were randomly assigned to two groups using random numbers and an allocation table to minimize bias. The allocation table was sealed by the investigator and kept sealed until the end of the study period. The controller, who was not involved in the study conduct, assigned the participants to the HC and placebo groups. Various tests were performed at the beginning of the study, week 4, and week 8 of the study period. Participants were instructed to consume the study foods and keep a daily diary during the study period.

### 2.4 Sampling

Blood, stool, and saliva samples were collected from the recruited subpopulation before and after the 8-week intake period to measure various parameters. PBMCs were collected from blood using Ficoll-Paque PLUS (Cytiva, Tokyo, Japan). The PBMCs were washed with PBS, immersed in cell bunkers, and stored frozen at -80°C. Fecal samples were collected using stool collection kits specified by Metagen, Inc. and TechnoSuruga Laboratory Co. Saliva samples were collected using Salisoft^®^ (SARSTEDT AG & Co. KG, Nümbrecht, Germany), according to the manufacturer’s instructions. Patients were instructed to only use toothbrushes without abrasives or rinses.

### 2.5 Endpoints

In this study, HC and placebo groups were established to evaluate the effects of HC administration on URTI symptom relief and immune activation. The primary endpoints were the upper respiratory symptom questionnaire, salivary sIgA, and fecal IgA. The questionnaire was based on the Wisconsin Upper Respiratory Symptom Survey (WURSS). Upper respiratory symptoms were recorded daily, and the cumulative number of days of onset and score were evaluated. Each symptom was scored on an 8-point scale, with “no symptoms” denoted by 0 and “very severe” denoted by 7. The cumulative number of onset days was calculated by summing the number of days with upper respiratory tract symptoms during the intake period for each participant and calculating the product of the sum and the relative intake ratio. The relative intake ratio was calculated by dividing the number of days of the intake period for each participant by 56 days, which was the number of days of the prescribed intake period. Salivary sIgA levels were measured using an ELISA kit (Yanaihara Institute Inc., Shizuoka, Japan). Fecal IgA levels were measured using an ELISA kit (TechnoSuruga Laboratory Co., Ltd., Shizuoka, Japan). Secondary endpoints included blood markers of inflammation, blood markers of stress, blood markers of immunity, the Profile of Mood States 2nd Edition (POMS-2), and the Medical Outcome Study Short-Form 36-Item Health Survey (SF-36^®^). Blood inflammatory marker levels, stress marker levels, and NK cell activity measured by the ^51^Cr release method were performed at LSI Medience Co.

### 2.6 Exposure culture of inactivated influenza virus on PBMCs

The measurement of blood immune markers and inactivated influenza virus exposure culture experiments were performed at the Kyoto Institute of Nutrition and Pathology, Inc. PBMCs were frozen and thawed in a water bath at 37°C and subsequently incubated in RPMI 1640. The medium was adjusted to 1.0 × 10^6^ cells/mL in RPMI 1640 medium, and 0.2 mL was seeded into 96-well cell culture plates. Inactivated influenza A H1N1 virus antigen (HyTest Ltd., Turku, Finland) was added at a concentration of 1.0 µg/mL. After incubation in a 5% CO2 incubator for 24 h, the culture supernatant was collected and stored frozen at -80°C. After culturing, cells were immersed in RNA-later and frozen at -80°C for storage. These samples were used for cytokine concentration analysis and gene expression analysis.

### 2.7 Analysis of cytokine concentrations

Regarding the measurement of various cytokine concentrations in the supernatant, frozen culture supernatants were thawed, and the concentrations of IL-2, IL-4, IL-6, IL-8, IL-10, TNFα, IFNγ, and IL-17A were measured using a commercial kit (Human Th1/Th2/Th17 Kit; BD Biosciences, Franklin Lakes, USA) with a flow cytometer (BD Accuri C6; BD Biosciences, Franklin Lakes, USA).

### 2.8 Gene expression analysis

Regarding gene expression analysis of cells after culture, total RNA was extracted using NucleoSpin^®^ RNA XS (MACHEREY-NAGEL GmbH & Co. KG, Düren, Germany). cDNA from total RNA solutions was synthesized using a reverse transcriptase (PrimeScript RT Reagent Kit; TAKARA, Shiga, Japan). Real-time PCR analysis was performed using a Rotor-Gene Q (Qiagen, Tokyo, Japan). Optimal primers and probes were designed using freely available online tools (https://primers.neoformit.com/), and these lists are shown in **Supplementary Table 2**. The relative expression levels of the mRNAs were calculated by the ΔΔCt Method. The amount of target relative to housekeeping mRNA (β-actin) was determined for comparison.

### 2.9 Analysis of organic acids in feces

Organic acid concentrations in feces were measured using gas chromatography (Metagen, Inc., Yamagata, Japan). After fecal derivatization, an internal standard for elution time correction was added to the feces for measurement. The column retention times, mass-to-charge ratios, and peak areas of the detected peaks were obtained, and quantification was performed by comparison with a calibration curve prepared using standards of known concentration.

### 2.10 Safety evaluation

Safety assessment was conducted based on the results of interviews during the study period, and adverse events were tabulated based on the contents of the participants’ diaries. In addition, abnormal variations in physical examination [weight, body mass index (BMI)], physical examination (blood pressure, pulse rate), and clinical laboratory tests (hematology, blood biochemistry, and urinalysis) were evaluated.

### 2.11 Statistical analysis

Among the participants for analysis who completed all the prescribed study schedule and study content, those who met the following criteria were excluded from the analyses: (1) those whose study food intake rate was less than 80%; (2) those whose behavior would undermine the reliability of the test results, such as missing diary records; (3) those who were found to meet the exclusion criteria after enrollment or who were found to be unable to comply with the restrictions during the study period; and (4) those who had obvious reasons for exclusion. A t-test was used for group comparisons of the participants’ backgrounds and various blood markers. A paired t-test was used for within-group comparisons to baseline values. The chi-squared test was used to compare male/female ratios between each group. The Mann–Whitney U test was used for between-group comparisons of upper respiratory tract infection symptom scores. sIgA concentrations and sIgA concentrations per unit of time were compared between groups using analysis of covariance corrected for pretest measurements, and a paired t-test was used for within-group comparisons to baseline values. The Mann–Whitney U test was used for between-group comparisons of various fecal organic acid concentrations, gene expression levels in the inactivated influenza virus exposure experiment, and various cytokine concentrations. Statistical analyses were performed using Excel Office365 (Microsoft, Washington, USA), SPSS ver. 27 (IBM, Tokyo, Japan), or R ver. 4.3.1 (R Core Team, Vienna, Austria). Plotting was performed with the R package ggplot2. Statistical significance was set at p < 0.05.

## 3 Results

### 3.1 Participants

**Figure 1:**
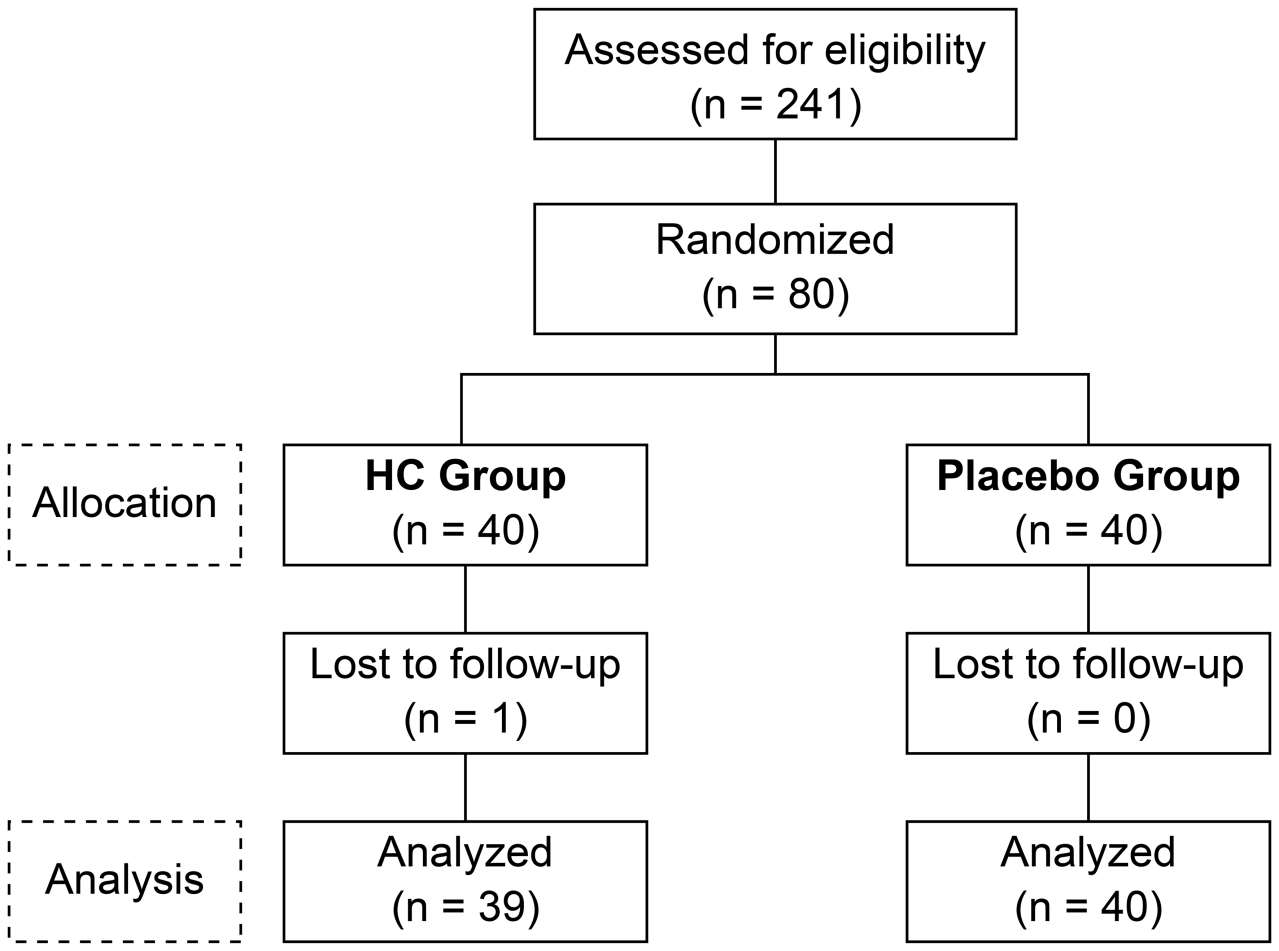
The consolidated standards of reporting trials flow diagram. HC: *Heyndrickxia coagulans* strain SANK70258.

The study flow chart course from participant selection and study diet allocation to analysis is shown in **Figure 1**. Of the 241 participants who participated in the pre-test, 80 met the inclusion criteria, did not violate the exclusion criteria, and were included. Of these 80 participants, one voluntarily withdrew from the study after the week 4 test. The background of the 79 participants in the efficacy analysis is shown in **Table 1**. Systolic blood pressure was significantly higher in the placebo group than in the HC group, with no significant differences in other parameters between the groups.

**Table 1:** Participant backgrounds in efficacy analysis.

		Study food	Placebo	p-value
		(HC)		
Number of participants		39	40	
Gender	Male	20	20	0.909
	Female	19	20	
Age		47.5 ± 10.0	48.8 ± 8.0	0.522
Height (cm)		165.65 ± 8.39	165.17 ± 8.99	0.808
Weight (kg)		58.03 ± 10.15	58.70 ± 9.70	0.768
BMI (kg/m2)		21.02 ± 2.44	21.42 ± 2.48	0.470
Systolic blood pressure (mmHg)		107.3 ± 11.2	114.6 ± 12.8	0.009
Diastolic blood pressure (mmHg)		72.9 ± 9.0	76.9 ± 9.1	0.051
Pulse rate (beats/minute)		74.5 ± 12.8	71.0 ± 11.0	0.194

All parameters are shown as the means ± the standard errors (except for number and gender). The Mann–Whitney U test was used for statistical comparisons of all variables, except for gender. The chi-squared test was used for statistical comparison of gender. HC, Heyndrickxia coagulans strain SANK70258; BMI, body mass index.

### 3.2 Questionnaire survey

The results of the URTI survey for each participant are shown in **Table 2**. Runny nose, nasal congestion, sneezing, and sore throat scores were significantly lower in the HC group than in the placebo group during the study period (p = 0.001, p = 0.006, p < 0.001, and p = 0.020, respectively). Furthermore, the cumulative number of days these symptoms were present showed a significant difference in the HC group compared to the placebo group (p = 0.008). No significant differences were observed in other symptoms. Regarding the POMS2, a longitudinal comparison from baseline to 8 weeks was conducted, and no significant differences were found for any of the items in either the HC or placebo groups (data not shown). Regarding the SF-36^®^, bodily pain improved significantly in the HC group after treatment, with no significant differences in the other parameters in either the HC or placebo groups (data not shown).

**Table 2:** Upper respiratory tract infection score during the study period.

Symptoms	Group	Score	p-value
Cumulative incidence number (days)	HC	13.1 ± 3.0	0.008
	Placebo	26.2 ± 3.7	
Runny nose	HC	13.1 ± 5.5	0.001
	Placebo	40.6 ± 7.8	
Stuffy nose	HC	11.8 ± 5.5	0.006
	Placebo	29.0 ± 6.6	
Sneezing	HC	5.7 ± 2.4	< 0.001
	Placebo	27.1 ± 6.1	
Sore throat	HC	0.7 ± 0.3	0.020
	Placebo	12.1 ± 5.9	
Faint voice	HC	0.4 ± 0.3	0.209
	Placebo	8.2 ± 5.2	
Cough	HC	1.3 ± 0.7	0.211
	Placebo	8.0 ± 4.5	
Headache	HC	2.9 ± 1.0	0.711
	Placebo	5.1 ± 1.7	
Chills	HC	1.8 ± 0.8	0.821
	Placebo	1.4 ± 0.6	
Lassitude	HC	4.8 ± 2.3	0.816
	Placebo	3.0 ± 1.1	
Articular pain	HC	2.1 ± 1.5	0.698
	Placebo	5.0 ± 4.6	
Phlegm	HC	2.2 ± 1.7	0.302
	Placebo	8.0 ± 4.0	
Fever	HC	0.1 ± 0.1	1.000
	Placebo	0.2 ± 0.2	

All parameters are shown as the means ± the standard errors. The Mann–Whitney U test was used for statistical comparisons.

HC: n = 39, placebo: n = 40.

HC: Heyndrickxia coagulans strain SANK70258.

### 3.3 Measurement of blood clinical markers

The clinical markers in the blood of each participant are shown in **Table 3**. The HC group had a significantly lower blood cortisol level at 8 weeks of treatment compared to baseline (p < 0.01), with no significant difference observed in the placebo group. IgG levels in each group were significantly lower at 8 weeks compared to baseline (p < 0.01). NK cell activity was significantly lower at 8 weeks in both the HC and placebo groups compared to baseline (p < 0.01). Decreased NK cell activity was observed in both groups; however, the decrease was greater in the placebo group than in the HC group, indicating that NK cell activity in the HC group was significantly higher than in the placebo group at both 4 and 8 weeks of treatment (p < 0.05).

**Table 3:** Serial changes in inflammation, stress, and immune markers in blood.

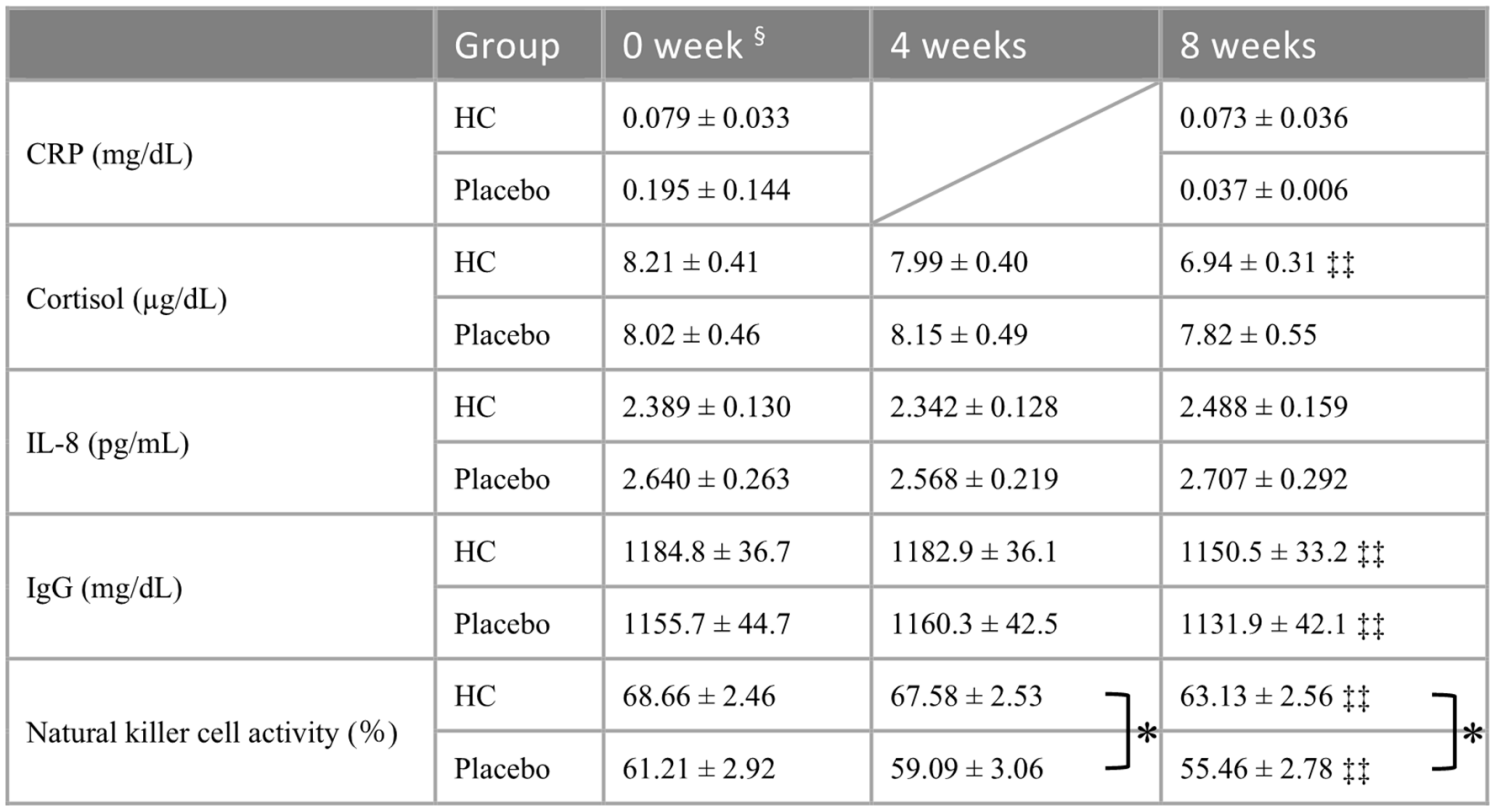

All parameters are shown as the means ± the standard errors.

‡ p < 0.05, ‡‡ p < 0.01; a paired t-test was used for within-group comparison to baseline. * p < 0.05; a t-test was used for comparison between groups at the same time point.

§ No significant differences were found in baseline comparisons between groups for each marker (p > 0.05).

HC: n = 39, placebo: n = 40.

HC: Heyndrickxia coagulans strain SANK70258.

### 3.4 IgA concentration measurement

**Figure 2:**
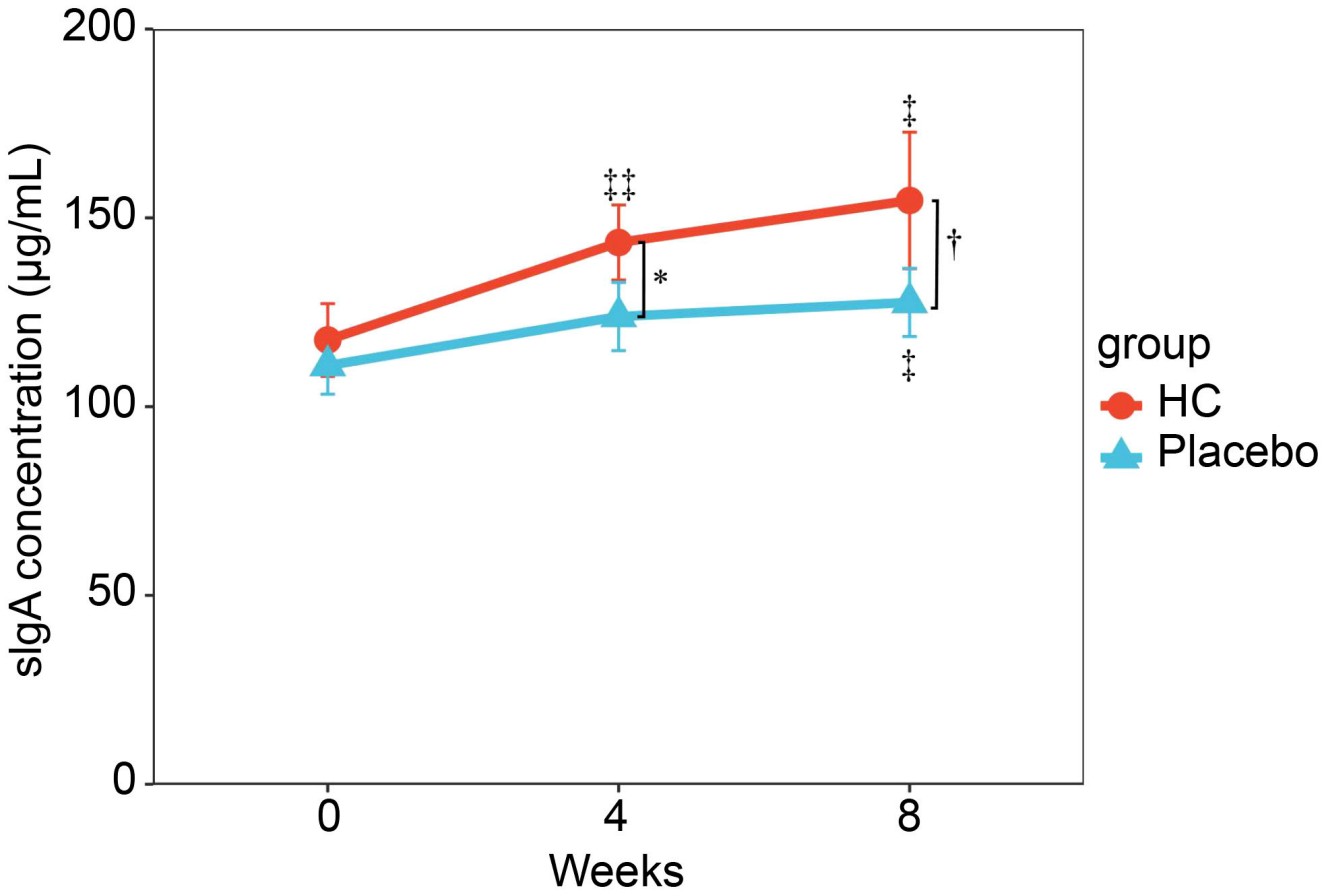
Serial changes in sIgA concentrations during the study period. Analysis of covariance (ANCOVA) adjusted for preliminary inspection measurements was performed between groups at the same time point. * p < 0.05, †p = 0.061. ‡ p < 0.05, ‡‡ p < 0.01; a paired t-test was used for within-group comparison to baseline. HC: n = 39, and placebo: n = 40. HC: *Heyndrickxia coagulans* strain SANK70258.

Changes in salivary sIgA concentration over time are shown in **Figure 2**, and sIgA concentration per unit of time and fecal IgA concentration are shown in **Supplementary Figure 1**. Salivary sIgA concentration was not significantly different between groups at baseline; however, it was significantly higher in the HC group at 4 weeks (p < 0.05) and tended to be higher in the HC group at 8 weeks (p = 0.061) compared to the placebo group. The HC group had significantly higher sIgA concentrations at 4 and 8 weeks of treatment compared to baseline (p < 0.01 and p < 0.05, respectively). In contrast, the placebo group showed significantly higher sIgA concentrations only at 8 weeks of treatment compared to baseline (p < 0.05). The sIgA concentration per unit of time also showed a similar trend. Fecal IgA concentrations were not significantly different between groups.

### 3.5 Fecal organic acid analysis

**Table 4** shows the results of the analysis of various organic acids in feces, with the changes in each concentration from baseline to 8 weeks. Significantly higher changes in formic, butyric, and valeric acids were observed in the HC group compared to the placebo group (p = 0.014, 0.013, and 0.038, respectively).

**Table 4:** Serial changes in organic acid concentrations during the study period.

	Group	0 week (µg/ml)	8 weeks (µg/ml)	Changes (µg/ml)	p-value
Formic acid	HC	1635 ± 257	2258 ± 558	624 ± 573	0.014
	Placebo	2595 ± 414	1658 ± 205	-937 ± 387	
Acetic acid	HC	163044 ± 14311	195007 ± 16771	31964 ± 12173	0.403
	Placebo	179842 ± 15749	197070 ± 17846	17228 ± 15926	
Propionic acid	HC	44102 ± 4086	48142 ± 4539	4040 ± 3674	0.282
	Placebo	54783 ± 5111	52085 ± 5160	-2698 ± 4138	
Isobutyric acid	HC	3637 ± 283	4060 ± 444	424 ± 459	0.256
	Placebo	4376 ± 361	4284 ± 364	-92 ± 475	
Butyric acid	HC	22105 ± 2255	23801 ± 2399	1696 ± 2178	0.013
	Placebo	24212 ± 2215	20107 ± 2540	-4105 ± 2555	
Isovaleric acid	HC	3424 ± 274	4724 ± 405	300 ± 421	0.160
	Placebo	4104 ± 392	4070 ± 419	-33 ± 497	
Valeric acid	HC	3939 ± 393	4256 ± 530	317 ± 420	0.038
	Placebo	5508 ± 595	4336 ± 422	-1172 ± 533	
Lactic acid	HC	2079 ± 749	15522 ± 8623	13443 ± 8563	0.282
	Placebo	1634 ± 604	1852 ± 518	218 ± 699	
Succinic acid	HC	7037 ± 2848	10711 ± 6447	3674 ± 7151	0.684
	Placebo	2257 ± 497	7812 ± 4981	5555 ± 4656	

All parameters are shown as the means ± the standard errors. The Mann–Whitney U test was used for statistical comparisons of the concentration of changes from baseline to 8 weeks.

HC: n = 39, placebo: n = 39 (One patient in the placebo group was excluded from the analysis because the patient did not comply with the fecal sampling procedure described in the protocol).

HC: Heyndrickxia coagulans strain SANK70258.

### 3.6 Effects of HC on the immune response to human influenza A/H1N1 virus

**Figure 3:**
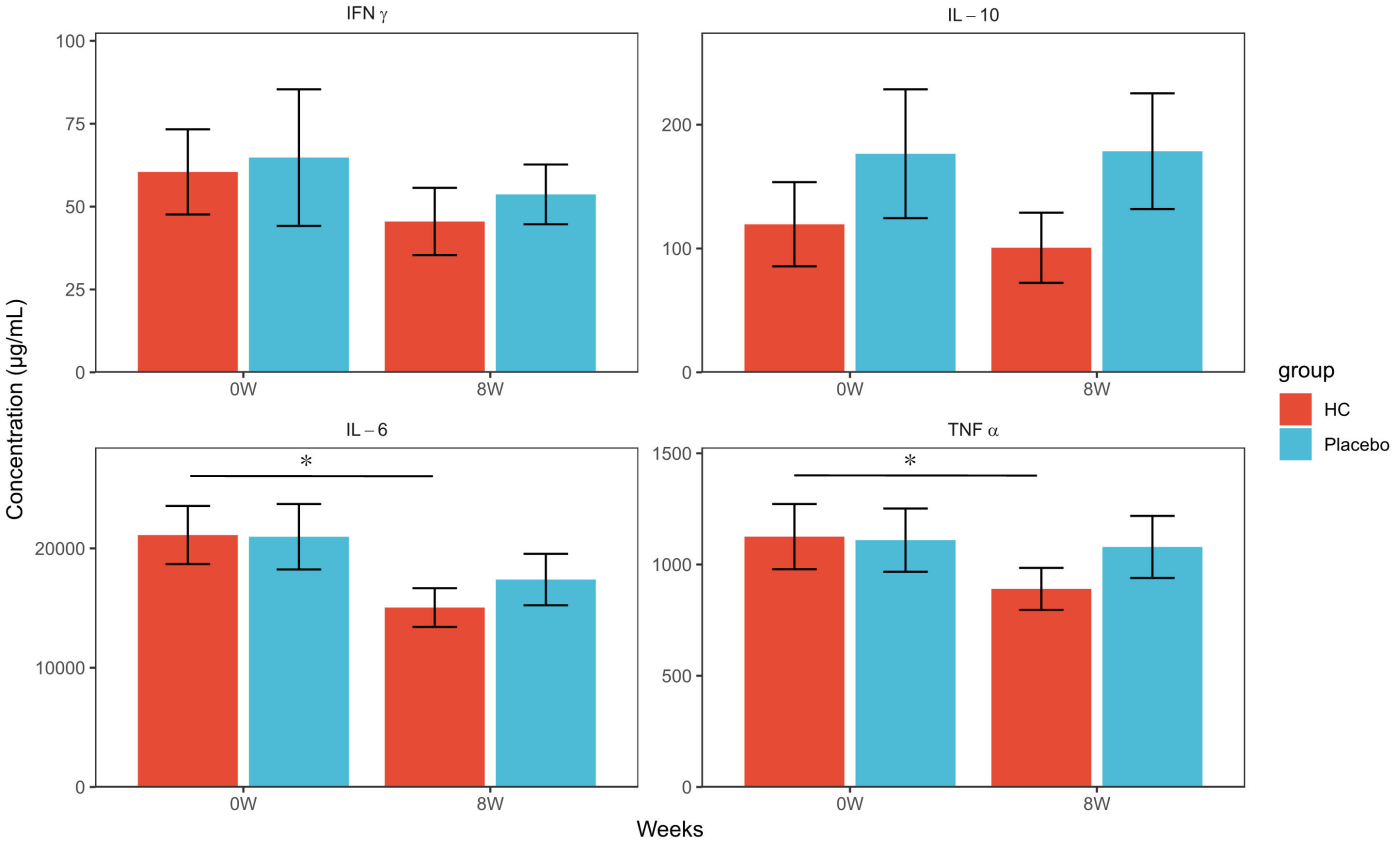
Cytokine production concentrations of PBMCs exposed to inactivated influenza virus at each time point. * p < 0.05; a paired t-test was used for within-group comparison to baseline. HC: n = 39, placebo: n = 40. HC: *Heyndrickxia coagulans* strain SANK70258.

The concentrations of various cytokines in the supernatant after cell culture were measured, and the results are shown in **Figure 3** and **Supplementary Figure 2**. PBMC at 8 weeks of intake showed significantly lower levels of IL-6 and TNFα production in the HC group compared to baseline (p < 0.05, p < 0.05, respectively), but no significant changes were observed in the placebo group. No significant differences in IFNγ and IL-10 levels were observed between the two groups. IL-2, IL-4, and IL-17A levels were measured in this study but were below the detection limit in all samples (data not shown).

**Figure 4:**
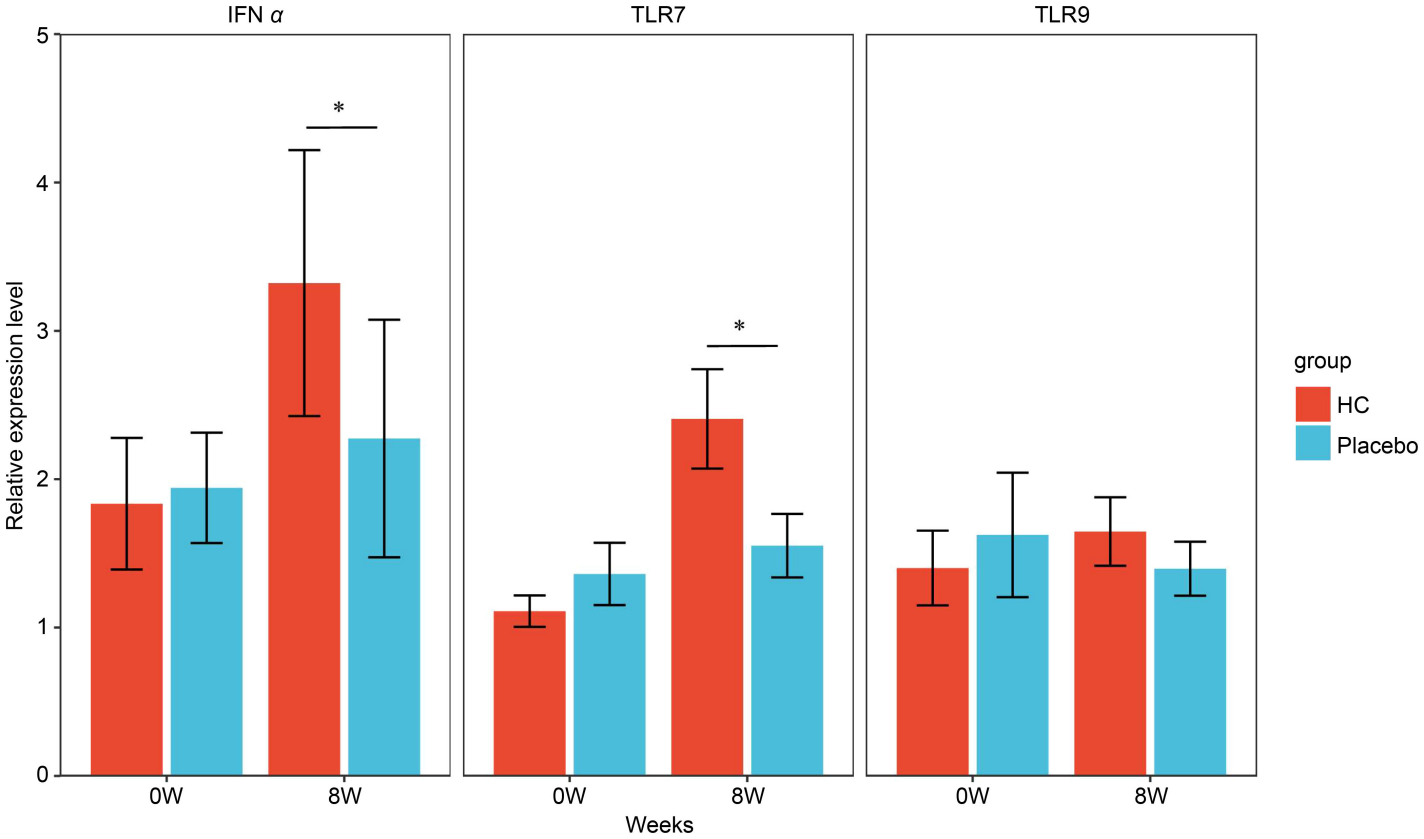
Gene expression levels in PBMCs exposed to inactivated influenza virus at each time point. * p < 0.05; the Mann–Whitney U test was used for statistical comparisons of each group. HC: n = 39, placebo: n = 40. HC: *Heyndrickxia coagulans* strain SANK70258.

The gene expression analysis of PBMC cells after the same exposure experiment is shown in **Figure 4**. No significant differences were observed in gene expression levels of IFNα and TLR7 between the groups at baseline; however, gene expression levels at 8 weeks were significantly higher in the HC group compared to the placebo group (p < 0.05). In addition, the gene expression level of CD304 was relatively low in all samples, particularly in the pre-exposure samples where the mean Ct value >40, and the measurement results varied widely (data not shown). Therefore, we performed a comparative analysis of CD304 gene expression level in samples with Ct values <40 to select appropriate samples for study (**Supplementary Figure 3**). No significant difference was observed in the CD304 gene expression level between groups at baseline; however, a trend towards higher CD304 gene expression level was observed in the HC group compared to the placebo group at 8 weeks (p = 0.05). CD303 gene expression analysis was performed in this study but was below the detection limit for all samples (data not shown).

### 3.7 Safety evaluation

An adverse event in this study was defined as any unwanted or unintended injury or symptom of injury or illness that occurred to the participant after the time of the pre-test. Among the total of 80 allocated patients, adverse events occurred in 9 out of 40 participants in the HC group, for a total of 22, and in 7 out of 40 participants in the placebo group, for a total of 23. These adverse events were determined by the treating physician to be unrelated to the study foods. In addition, one case of dizziness and thought disorder occurred in the HC group. However, after a comprehensive evaluation of these symptoms using interviews, the treating physician judged that there were no safety issues because the symptoms were considered to have a strong mental component. The measurements of the blood and urine tests are shown in **Supplementary Tables 3** and **4**, respectively. Several items showed significant changes at 8 weeks compared to the pre-treatment values; however, they were all considered to be minor changes within the reference values and were judged to be clinically acceptable by the treating physician. **Supplementary Table 5** shows the changes in the physical examinations. Several items in physical examinations showed significant changes at 8 weeks compared to the pre-treatment values; however, they were all considered to be minor changes within the reference values and were judged to be clinically acceptable by the treating physician.

## 4 Discussion

The results of this study showed that URTI-related scores, such as runny nose and sore throat scores, and the cumulative number of days of these symptoms during the intervention period were significantly lower in the HC group than in the placebo group (**Table 3**), suggesting that HC may have a mitigating effect on URTI symptoms. In addition, salivary sIgA concentrations were significantly higher in the HC group than in the placebo group at 4 weeks, and this trend was maintained until 8 weeks (**Figure 2**). Moreover, the NK cell activity was also maintained at high levels in the HC group compared to the placebo group until 8 weeks (**Table 3**). Previous *in vivo* and *in vitro* studies report that *H. coagulans* administration increases NK cell activation and IgA production in the intestine, spleen, and bone marrow (19, 23). Furthermore, human clinical research on a different strain has shown that *H. coagulans* ingestion increases NK cell activity (20). Probiotic-induced NK cell activity and salivary sIgA levels are well known to be involved in the alleviation of URTI (8–11). These previous reports are in line with the results of this study.

Furthermore, we performed an exposure culture assay of inactivated influenza virus on PBMCs isolated from the blood of participants in the HC and placebo groups to verify the detailed mechanism of this immune activation. Gene expression analysis in PBMCs after culture showed that the expression level of the IFNα gene was significantly higher in the HC group than in the placebo group (**Figure 3**). IFNα is one of the representative cytokines produced and induced in large amounts by pDC in the early stages of viral and microbial infection and has been reported to have antiviral and antimicrobial activity (24). IFNα has been reported to contribute to the induction of NK cell activity and IgA production (25, 26), and the increased sIgA levels and suppression of decrease in NK cell activity observed in this study may be due to increased IFNα production by pDC. The gene expression level of the TLR7 gene was also significantly higher in the HC group than in the placebo group after the end of incubation (**Figure 4**). TLR7, which recognizes viral RNA, is present in pDC endosomes, and recognition of viral RNA by this receptor during a viral infection induces an antiviral infection cascade through the production of IFNα and other factors (27). TLR7 has been reported to be most highly expressed in pDCs among the cells constituting PBMCs (28). Furthermore, Siegal et al. reported that in HSV exposure studies performed in various cellular fractions of PBMCs. IFNα production and gene expression were specifically enhanced in the pDC fraction of PBMCs, whereas IFNα was hardly enhanced in the B-cell, T-cell, and monocyte fractions, suggesting that IFNα production in PBMCs is primarily in pDCs (24). Based on these reports, the fact that IFNα and TLR7 genes are upregulated in tandem in the HC group in this study strongly suggests activation of pDC in PBMCs by HC. Our hypothesis was also supported by the finding that CD304, one of the surface antigens of pDC, showed a trend towards higher gene expression levels after incubation in the HC group compared to the placebo group (p=0.05). Based on the results of the culture study and previous reports, HC administration may have induced pDC activation *in vivo*, leading to early and strong induction of immune responses, such as NK cell activation and increased sIgA levels associated with IFNα upregulation at the time of viral infection. In addition, type I IFNs, including IFNα produced by pDC, are known to induce several antiviral factors that limit viral replication and transmission (29), suggesting the existence of other mechanisms that contribute to the prevention of viral infection besides the above hypothesis. Previous research suggests that the DNA eluted by phagocytosis of pDC can act as a ligand for the activation of pDC in *L. lactis* ssp. *lactis* JCM5805 and that cell wall components also act as ligands (30). Further studies are required to clarify the mechanism by which HC activates pDC; nevertheless, a report on co-culture of bone marrow-derived dendritic cells with HC indicated that cytokine induction by HC is suppressed when the expression of PAMP receptors, which recognize peptidoglycan-related components, is decreased in dendritic cells (19). pDC has the same receptor; therefore, the presence of HC-specific peptidoglycan and other bacterial components may induce immune activation in the host, without relying on the effects of HC regarding increased intestinal organic acid concentration or improved intestinal microbiota. However, since this study was conducted in PBMCs, in the future, we would like to evaluate whether the same trend is observed by examining gene expression levels in pDCs alone.

The increase in the concentration of various organic acids in feces was evaluated from baseline to 8 weeks, and the increase in butyric acid was found to be significantly higher in the HC group than in the placebo group (**Table 4**). A study evaluating human fecal culture demonstrated that HC enhances butyrogenesis in the gut microbiome in collaboration with lactate-utilizing butyrate-producing bacteria, such as *Lachnospiraceae*, which is consistent with the results of our study (31–33). Furthermore, the concentrations of proinflammatory cytokines in the culture supernatant were compared in the PBMC exposure experiment with the inactivated influenza virus, and no significant differences were observed in the concentrations of various cytokines in PBMCs before and after the intervention period in the placebo group; however, TNFα and IL-6 concentrations in the HC group were significantly lower in PBMCs at 8 weeks compared to baseline (**Figure 3**). Butyric acid is recognized by GPR109A, a receptor on dendritic cells and macrophages, and is known to suppress excessive inflammation by inducing anti-inflammatory effects, such as Treg activation (34). In addition, a meta-analysis of athletes suggested that one of the points of action of probiotics in alleviating URTI may be the suppression of IL-6 and TNFα (35). In addition to the pDC-mediated immunostimulatory mechanism of HC ingestion considered in this study, HC may have alleviated URTI symptoms by increasing intestinal butyrate concentration through an increase in butyrate-producing bacteria, which suppresses excessive inflammatory responses during viral infection. Butyrate produced by gut bacteria has been reported to have an inhibitory effect on the development of cytokine storms caused by COVID-19 infection (36). In this study, HC intake was considered to increase butyrate production in the gut, which may suppress the production of cytokines such as IL-6, suggesting that HC may contribute to cytokine storm suppression. In addition, HC may have the potential to suppress the excessive production of IL-8 due to viral infection. In the same exposure experiment, cell culture samples without exposure to inactivated influenza virus were prepared as negative controls, and IL-8 production was compared at each time point. IL-8 levels were significantly higher in samples with inactivated influenza virus than in the negative control in the placebo group at 8 weeks; however, no significant difference was observed between samples with and without inactivated influenza virus in the HC group (**Supplementary Figure 2**). IL-8, similar to IL-6, is another cytokine involved in the cytokine storm, and the results of IL-8 levels in this study also support our hypothesis (37).

Notably, blood cortisol concentration was specifically and significantly reduced only in the HC group at 8 weeks compared to baseline (**Table 4**), and the increase in blood cortisol concentration tended to be lower in the HC group than in the placebo group (p=0.053, data not shown). Cortisol production is strongly induced when blood IL-6 levels are high, and the suppressive effect of HC on IL-6 levels during viral infection in this study may have reduced cortisol levels (38). Furthermore, cortisol is reported to decrease NK cell activity (39). In this study, NK cell activity was increased, along with the decrease in cortisol after HC intake, which is consistent with previous reports. Cortisol is widely used as a marker to assess stress; therefore, HC may also contribute to stress reduction. However, this requires verification in further studies.

This study had some limitations. First, the number of pDC cells was not evaluated since fluorescence-activated cell sorting was not performed in the pDC experiments. In addition, the anti-inflammatory effect of butyrate produced by butyrate-producing bacteria, which is increased by HC, may contribute to the alleviation of URTI symptoms; however, the dynamics of butyrate-producing bacteria were not evaluated because intestinal microbiota analysis was not performed. These factors should be evaluated in future studies.

In this study, we found that HC has immune modulation properties through pDC activation, which is similar to the previously reported effects of lactic acid-producing and acetic acid-producing bacteria. In addition, HC, a live bacterium, has been suggested to be effective in modifying the composition of the intestinal microbiota and intestinal organic acids, which are effects believed to be unique to probiotics.

## Data availability statement

The original contributions presented in the study are included in the article/**Supplementary Material**. Further inquiries can be directed to the corresponding author.

## Ethics statement

This study was conducted in accordance with the tenets of the Declaration of Helsinki, was approved by the Research Ethics Committee of the Medical Station Clinic (approval date: December 24, 2020; approval number: 20000022), and was registered and published in the UMIN clinical trial registration system (clinical trial registration number: UMIN000042937). The study participants received a full explanation of the study contents from the investigator and provided voluntary written consent to participate. The studies were conducted in accordance with the local legislation and institutional requirements. The participants provided their written informed consent to participate in this study.

## Author contributions

MA: Conceptualization, Data curation, Formal analysis, Investigation, Methodology, Software, Validation, Visualization, Writing – original draft, Writing – review & editing. NT: Data curation, Formal analysis, Investigation, Methodology, Software, Validation, Writing – review & editing. KM: Data curation, Formal analysis, Methodology, Validation, Writing – review & editing. YA: Conceptualization, Investigation, Methodology, Writing – review & editing. SS: Data curation, Investigation, Methodology, Validation, Writing – review & editing. AS: Investigation, Methodology, Writing – review & editing. NU: Conceptualization, Funding acquisition, Investigation, Project administration, Resources, Supervision, Writing – review & editing. RY: Conceptualization, Data curation, Formal analysis, Funding acquisition, Investigation, Project administration, Writing – review & editing.
