# Supplementary material for: Heyndrickxia coagulans strain SANK70258 suppresses symptoms of upper respiratory tract infection via immune modulation: a randomized, double-blind, placebo-controlled, parallel-group, comparative study
Source: Front Immunol. 2024 Jun 17;15:1389920. doi: 10.3389/fimmu.2024.1389920 (PMC11218553; doi:10.3389/fimmu.2024.1389920)
Supplement: Supplementary file 1 [file DataSheet_1.docx]

Supplementary Material

# Supplementary Figures


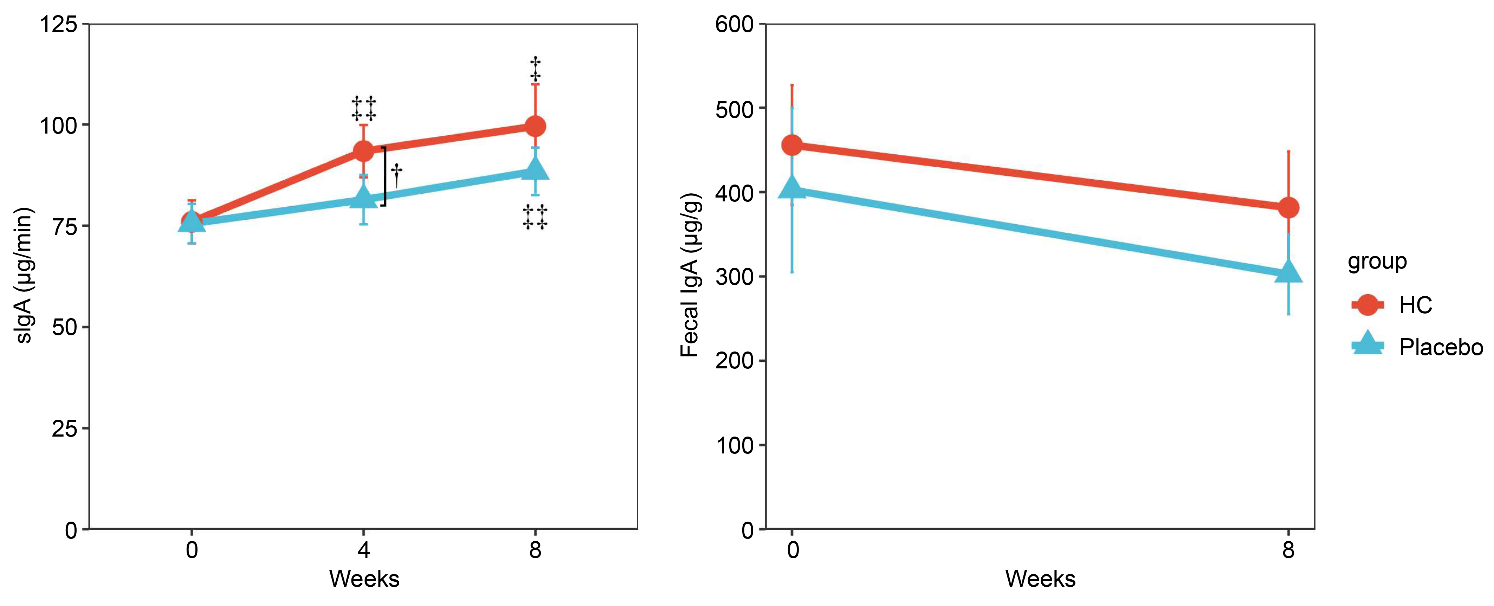


**Figure S1. Serial changes in sIgA concentrations per minute and fecal IgA concentrations during the study period**

Analysis of covariance (ANCOVA) adjusted for preliminary inspection measurements was performed between groups at the same time point. † p = 0.066

‡ p < 0.05, ‡‡ p < 0.01 a paired t-test was used for within-group comparison to baseline.

A t-test was used to compare fecal IgA between groups; however, no significant differences were found.

sIgA; HC: n = 39, placebo: n = 40.

Fecal IgA; HC: n = 39, and placebo: n = 39 (One patient in the placebo group was excluded from the analysis because the patient did not comply with the fecal sampling procedure described in the protocol.).

HC: *Heyndrickxia coagulans* strain SANK70258


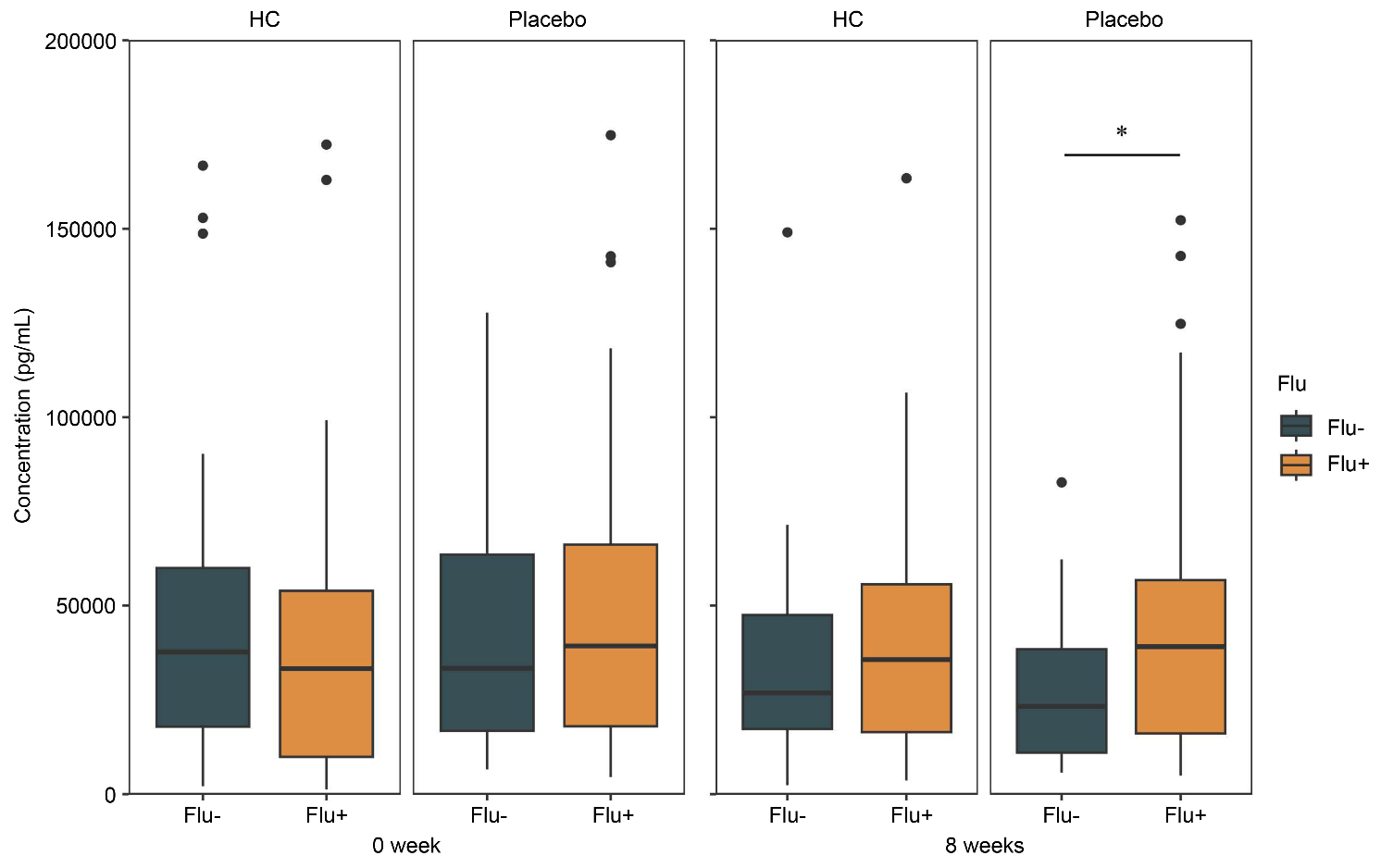


**Figure S2. IL-8 production concentrations of PBMCs exposed or not exposed to inactivated influenza virus at each time point**

* p < 0.05 The Mann–Whitney U test was used for statistical comparisons of each group

HC: n = 39, placebo: n = 40.

HC: *Heyndrickxia coagulans* strain SANK70258


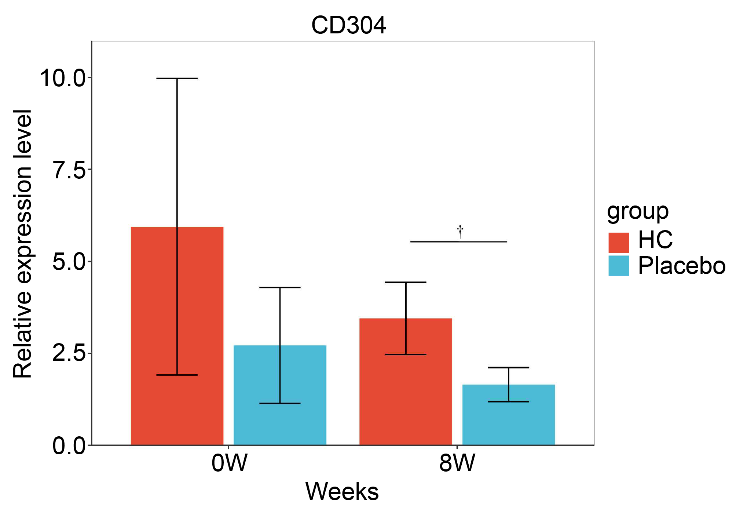


**Figure S3. Gene expression levels in PBMCs exposed to inactivated influenza virus at each time point**

†p = 0.05 The Mann–Whitney U test was used for statistical comparisons of each group

0W; HC: n = 5, placebo: n = 7.

8W; HC: n = 18, placebo: n = 25.

HC: *Heyndrickxia coagulans* strain SANK70258

**2 Supplementary Tables**

**Table S1-1. Composition of study foods**

HC: *Heyndrickxia coagulans* strain SANK70258

|  | Study food (HC) | Placebo |
| --- | --- | --- |
| Form | Hard capsule | Hard capsule |
| Raw materials | Dextrin, Heyndrickxia coagulans SANK70258, Cellulose, HPMC, Calcium stearate, Silicon dioxide | Dextrin, Cellulose, HPMC, Calcium stearate, Silicon dioxide |
| Functional ingredient | 67 mg (1 billion CFU)/grain | 0 mg/grain |
| Manufacture Date | 15-Jan-21 | 15-Jan-21 |
| Storage Method | Store in a dark place at room temperature, out of direct sunlight. | |
| Study Food Donors | Mitsubishi Chemical Corporation | |

**Table S1-2. Nutritional Composition of Study Foods**

HC: *Heyndrickxia coagulans* strain SANK70258

| Nutrition Facts | | Study food (HC) | Placebo |
| --- | --- | --- | --- |
| Energy | (kcal) | 1.051 | 0.933 |
| Protein | (g) | 0 | 0 |
| Lipid | (g) | 0 | 0 |
| Carbohydrate | (g) | 262.7 | 233.2 |
| Salt equivalent | (g) | 0 | 0 |

**Table S2. List of analyzed genes and used primers and probes**

The listed probe numbers indicate the product numbers of the Universal Probe Library set and Human and Extension sets sold by Roche Diagnostics (Tokyo, Japan).

| Gene | Accession No. | Universal Probe Library No. | Primers | Sequences |
| --- | --- | --- | --- | --- |
| β-actin | NM_001101.3 | #64 | Forward | CCAACCGCGAGAAGATGA |
|  |  |  | Reverse | CCAGAGGCGTACAGGGATAG |
| IFNα | NM_024013.3 | #64 | Forward | GCTCTCTGGGCTGTGATCTC |
|  |  |  | Reverse | TCTGCTCATTTGTGCCAGGA |
| TLR7 | NM_016562.4 | #10 | Forward | GAGGCTGAGGCAGGAGAATC |
|  |  |  | Reverse | CATTGCAGTGGCGTGATCTC |
| TLR9 | NM_017442.4 | #8 | Forward | CAACAACCTCACTGTGGTGC |
|  |  |  | Reverse | GACGATGCGGTTGTAGGACA |
| CD303 | NM_203503.2 | #124 | Forward | TGTTGGCCAGGCTAGTCTTG |
|  |  |  | Reverse | ATCTCAGCACTTTGGGAGGC |
| CD304 | NM_001024629.3 | #104 | Forward | GATGCCTGAAAACATCCGCC |
|  |  |  | Reverse | CCCCAGGTCTATTTGGAGCC |

**Table S3. Safety assessment (blood examination)**

* p < 0.05, ** p < 0.01 Paired t-test for within-group comparison to baseline.

HC: n = 39, and placebo: n = 40.

HC: *Heyndrickxia coagulans* strain SANK70258

|  |  |  |  |  |  |  |  |  |
| --- | --- | --- | --- | --- | --- | --- | --- | --- |
|  | Reference value | Group | Preliminary inspection | | | 8 weeks | | |
| White blood cell count (cells/µL) | 3300-9000 | HC | 4897 | ± | 1022 | 4833 | ± | 1204 |
|  |  | Placebo | 4990 | ± | 1241 | 5043 | ± | 1289 |
| Red blood cell count (×10^4^cells/µL) | M：430-570 | HC | 473.8 | ± | 36.0 | 474.1 | ± | 35.4 |
|  |  | Placebo | 493.9 | ± | 41.5 | 495.4 | ± | 45.3 |
|  | F：380-500 | HC | 440.8 | ± | 34.5 | 439.6 | ± | 34.4 |
|  |  | Placebo | 431.5 | ± | 30.4 | 426.8 | ± | 33.6 |
| Hemoglobin (g/dL) | M：13.5-17.5 | HC | 15.04 | ± | 0.98 | 14.84 | ± | 1.02 |
|  |  | Placebo | 15.39 | ± | 1.09 | 15.23 | ± | 1.09 |
|  | F：11.5-15.0 | HC | 13.25 | ± | 1.3 | 12.96 | ± | 1.21 |
|  |  | Placebo | 13.46 | ± | 0.7 | 13.14 | ± | 0.71* |
| Hematocrit (%) | M：39.7-52.4 | HC | 45.79 | ± | 3.11 | 46.29 | ± | 2.69 |
|  |  | Placebo | 46.63 | ± | 3.26 | 47.08 | ± | 3.26 |
|  | F：34.8-45.0 | HC | 41.26 | ± | 3.86 | 41.46 | ± | 3.67 |
|  |  | Placebo | 41.36 | ± | 2.07 | 41.26 | ± | 1.74 |
| Platelet count (×104cells/µL) | 14.0-34.0 | HC | 26.06 | ± | 6.29 | 25.45 | ± | 6.40 |
|  |  | Placebo | 25.82 | ± | 5.01 | 24.99 | ± | 5.14* |
| Total protein (g/dL) | 6.7-8.3 | HC | 7.08 | ± | 0.39 | 7.17 | ± | 0.35 |
|  |  | Placebo | 7.08 | ± | 0.41 | 7.2 | ± | 0.41* |
| Albumin (g/dL) | 3.8-5.2 | HC | 4.38 | ± | 0.28 | 4.47 | ± | 0.25* |
|  |  | Placebo | 4.40 | ± | 0.27 | 4.54 | ± | 0.33** |
| Total bilirubin (mg/dL) | 0.2-1.2 | HC | 0.88 | ± | 0.29 | 0.84 | ± | 0.32 |
|  |  | Placebo | 0.82 | ± | 0.27 | 0.84 | ± | 0.31 |
| direct bilirubin (mg/dL) | 0.0-0.2 | HC | 0.11 | ± | 0.07 | 0.12 | ± | 0.06 |
|  |  | Placebo | 0.11 | ± | 0.05 | 0.13 | ± | 0.06* |
| Indirect bilirubin (mg/dL) | 0.2-1.0 | HC | 0.78 | ± | 0.25 | 0.72 | ± | 0.29 |
|  |  | Placebo | 0.71 | ± | 0.23 | 0.71 | ± | 0.26 |
| AST (U/L) | 10-40 | HC | 20.6 | ± | 5.0 | 20.0 | ± | 4.8 |
|  |  | Placebo | 20.9 | ± | 4.3 | 20.2 | ± | 5.3 |
| ALT (U/L) | 5-45 | HC | 17.6 | ± | 6.5 | 16.5 | ± | 6.3 |
|  |  | Placebo | 17.6 | ± | 7.0 | 16.8 | ± | 6.8 |
| γ-GTP (U/L) | M：0-80 | HC | 27.2 | ± | 14.3 | 27.3 | ± | 17.6 |
|  |  | Placebo | 26.8 | ± | 17.9 | 25.7 | ± | 14.3 |
|  | F：0-30 | HC | 13.9 | ± | 5.5 | 13.8 | ± | 5.4 |
|  |  | Placebo | 21 | ± | 10 | 19.6 | ± | 11.8 |
| LD (U/L) | 124-222 | HC | 177.4 | ± | 24.8 | 167.7 | ± | 28.4** |
|  |  | Placebo | 179.8 | ± | 26.7 | 172.3 | ± | 24.4* |
| ALP (U/L) | 38-113 | HC | 64.1 | ± | 14.9 | 63.9 | ± | 13.3 |
|  |  | Placebo | 65.1 | ± | 19.9 | 64.7 | ± | 20.9 |
| Urea nitrogen (mg/dL) | 8.0-20.0 | HC | 13.31 | ± | 4.07 | 13.6 | ± | 4.07 |
|  |  | Placebo | 11.57 | ± | 3.05 | 13.27 | ± | 4.06** |
| Creatine (mg/dL) | M：0.61-1.04 | HC | 0.83 | ± | 0.09 | 0.84 | ± | 0.09 |
|  |  | Placebo | 0.85 | ± | 0.12 | 0.88 | ± | 0.12* |
|  | F：0.47-0.79 | HC | 0.63 | ± | 0.09 | 0.67 | ± | 0.09** |
|  |  | Placebo | 0.60 | ± | 0.09 | 0.65 | ± | 0.08** |
| Uric acid (mg/dL) | M：3.8-7.0 | HC | 5.22 | ± | 1.1 | 5.15 | ± | 1.21 |
|  |  | Placebo | 5.56 | ± | 1.08 | 5.76 | ± | 1.03 |
|  | F：2.5-7.0 | HC | 4.40 | ± | 1.00 | 4.04 | ± | 0.76* |
|  |  | Placebo | 3.97 | ± | 0.76 | 4.18 | ± | 0.94* |
| Total cholesterol (mg/dL) | 120-219 | HC | 209.7 | ± | 25.8 | 205.6 | ± | 28.3 |
|  |  | Placebo | 209.7 | ± | 27.8 | 205.1 | ± | 29.5 |
| Triglycerides (mg/dL) | 30-149 | HC | 72.2 | ± | 42.4 | 73.4 | ± | 50.9 |
|  |  | Placebo | 76.2 | ± | 37.0 | 68.0 | ± | 27.2 |
| HDL cholesterol (mg/dL) | M：40-85 | HC | 65.4 | ± | 15.2 | 64.6 | ± | 15.3 |
|  |  | Placebo | 69.0 | ± | 14.0 | 67.7 | ± | 13.7 |
|  | F：40-95 | HC | 83.3 | ± | 19.0 | 81.6 | ± | 20.2 |
|  |  | Placebo | 88.8 | ± | 18.4 | 90.3 | ± | 20.9 |
| LDL cholesterol (mg/dL) | 65-139 | HC | 120.8 | ± | 19.8 | 117.2 | ± | 23.5 |
|  |  | Placebo | 115.7 | ± | 22.4 | 111.2 | ± | 25.9 |
| Na (mEq/L) | 137-147 | HC | 140.8 | ± | 1.8 | 141.3 | ± | 1.5 |
|  |  | Placebo | 140.9 | ± | 1.8 | 141.1 | ± | 1.6 |
| K (mEq/L) | 3.5-5.0 | HC | 4.38 | ± | 0.29 | 4.38 | ± | 0.45 |
|  |  | Placebo | 4.56 | ± | 0.39 | 4.40 | ± | 0.36* |
| Cl (mEq/L) | 98-108 | HC | 102.8 | ± | 1.9 | 103.1 | ± | 1.7 |
|  |  | Placebo | 103.0 | ± | 1.8 | 102.9 | ± | 2.3 |
| Blood glucose (mg/dL) | 70-109 | HC | 86.3 | ± | 6.8 | 85.1 | ± | 7.1 |
|  |  | Placebo | 85.8 | ± | 7.5 | 84.3 | ± | 8.0 |
|  |  |  |  |  |  |  |  |  |

**Table S4. Safety assessment (urine examination)**

HC: n = 39, placebo: n = 40.

HC: *Heyndrickxia coagulans* strain SANK70258

|  |  |  |  |  |  |  |  |  |  |  |  |  |
| --- | --- | --- | --- | --- | --- | --- | --- | --- | --- | --- | --- | --- |
|  | Group | Preliminary inspection | | | | |  | 8 weeks | | | | |
|  |  | （-） | （±） | （+） | （2+） | （3+） |  | （-） | （±） | （+） | （2+） | （3+） |
| Urine protein | HC | 39 | 0 | 0 | 0 | 0 |  | 38 | 1 | 0 | 0 | 0 |
| （Qualitative） | Placebo | 38 | 2 | 0 | 0 | 0 |  | 38 | 1 | 0 | 0 | 1 |
| Blood glucose | HC | 39 | 0 | 0 | 0 | 0 |  | 39 | 0 | 0 | 0 | 0 |
| （Qualitative） | Placebo | 40 | 0 | 0 | 0 | 0 |  | 39 | 0 | 0 | 1 | 0 |
| Blood in urine | HC | 35 | 4 | 0 | 0 | 0 |  | 37 | 0 | 1 | 1 | 0 |
| （Qualitative） | Placebo | 38 | 0 | 0 | 0 | 2 |  | 39 | 0 | 0 | 0 | 1 |
|  |  |  |  |  |  |  |  |  |  |  |  |  |

**Table S5 Safety assessment (physical examination)**

* p < 0.05, ** p < 0.01 A paired t-test was used for within-group comparison to baseline.

HC: n = 39, placebo: n = 40.

HC: *Heyndrickxia coagulans* strain SANK70258; BMI: body mass index.

|  |  |  |  |  |  |  |  |  |  |  |
| --- | --- | --- | --- | --- | --- | --- | --- | --- | --- | --- |
|  | Group | Preliminary inspection | | | 4 weeks | | | 8 weeks | | |
| Weight (kg) | HC | 57.78 | ± | 10.35 | 57.69 | ± | 10.22 | 57.47 | ± | 9.96 |
|  | Placebo | 58.41 | ± | 10.16 | 58.24 | ± | 10.07 | 58.06 | ± | 9.94* |
| BMI (kg/m2) | HC | 20.92 | ± | 2.44 | 20.89 | ± | 2.40 | 20.81 | ± | 2.32 |
|  | Placebo | 21.3 | ± | 2.57 | 21.25 | ± | 2.54 | 21.18 | ± | 2.52* |
| Body fat ratio (%) | HC | 23.24 | ± | 6.49 | 23.21 | ± | 6.55 | 23.26 | ± | 6.74 |
|  | Placebo | 24.63 | ± | 6.30 | 24.34 | ± | 6.49 | 24.33 | ± | 6.47 |
| Muscle mass (kg) | HC | 41.8 | ± | 8.20 | 41.76 | ± | 8.17 | 41.57 | ± | 8.00 |
|  | Placebo | 41.53 | ± | 8.07 | 41.57 | ± | 8.17 | 41.45 | ± | 8.11 |
| Lean body weight (kg) | HC | 44.33 | ± | 8.60 | 44.27 | ± | 8.57 | 44.07 | ± | 8.38 |
|  | Placebo | 44.02 | ± | 8.52 | 44.06 | ± | 8.62 | 43.93 | ± | 8.54 |
| Systolic blood | HC | 108.4 | ± | 11.6 | 105.9 | ± | 10.8* | 106.4 | ± | 12.3* |
| Pressure (mmHg) | Placebo | 114.2 | ± | 11.8 | 110.9 | ± | 13.4* | 113 | ± | 12.3 |
| Diastolic blood | HC | 73.5 | ± | 8.9 | 73.4 | ± | 7.8 | 72.9 | ± | 9.6 |
| pressure (mmHg) | Placebo | 76.9 | ± | 9.4 | 75.6 | ± | 9.8 | 77.7 | ± | 8.3 |
| Pulse rate (beats/minute) | HC | 74.0 | ± | 12.7 | 74.1 | ± | 10.4 | 72.8 | ± | 10.9 |
|  | Placebo | 72.3 | ± | 9.5 | 72.0 | ± | 9.7 | 72.6 | ± | 9.5 |
|  |  |  |  |  |  |  |  |  |  |  |
